# A Bitter Pill

**Published:** 1891-11

**Authors:** 


—A Bitter Pill. — The *Southern Journal of Homeopathy* sends out a wail of disappointment, as follows :

"Governor Hogg, of Texas, has appointed Dr. Geo. W. Barker, of Waco, Superintendent of the Southwestern Asylum for the Insane, at San Antonio. It was hoped, against reasonable hope, however, that homeopathy might secure this new institution for the exemplification of her methods in the treatment of the mentally afflicted, but our time is not yet come. Patience, perseverance and perpetual pounding at the prejudices of the pill peddlers of this province will eventually win for us the recognition which is justly our due."

Observe the "p's." "Peter Piper picked a peck of pickled peppers," but he was not as much disappointed as was this little fellow, who had "hoped against reasonable hope" of picking this plum.

[Dr. W. L. Barker is the appointee.—Ed.]

---

It seems like boasting, to publish the pleasant things said about the Journal, but it is only human nature ; we all like approbation. We hope, therefore, we will be pardoned for reproducing the following ; it makes us feel that our efforts are not in vain ; and it may induce others to inquire for the Journal, who have never seen it.

The *Mississippi Medical Monthly*, published at Meridian, Miss., says: "We think you have the most readable journal published in the South."

"I cannot do without the Journal. It improves every year, and you deserve a great deal of credit for bringing it up to its present high standard. It is just what the Texas M. D.'s need,—a journal fearless and outspoken for the right, on all subjects of interest to the profession.

"H. C. G., M. D., Oakland, Texas."

---

Original Articles, essays on medical or surgical subjects, reports of cases, etc., are solicited. Secretaries of Medical societies are invited to send us the best of the papers read before their respective societies.

---

—Wanted—Back numbers of The Journal, from July, '91, to date. Cash, or credit on account, will be given for them.

